# Aggregation of a Parkinson’s Disease-Related Peptide: When Does Urea Weaken Hydrophobic Interactions?

**DOI:** 10.1021/acschemneuro.2c00169

**Published:** 2022-05-26

**Authors:** N. Galamba

**Affiliations:** BioISI—Biosystems and Integrative Sciences Institute, Faculty of Sciences of the University of Lisbon, C8, Campo Grande, 1749-016 Lisbon, Portugal

**Keywords:** neurodegenerative diseases, protein aggregation, α-synuclein, hydrophobic effect, proteinopathies, synucleinopathies

## Abstract

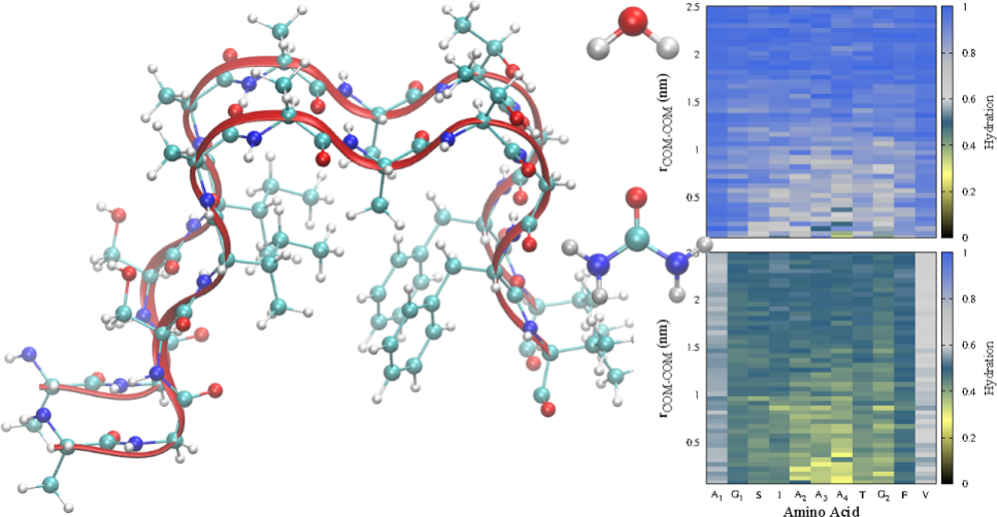

While the exact cause of neurodegenerative diseases such as Alzheimer’s disease and Parkinson’s disease is not completely understood, compelling evidence implicates the aggregation of specific proteins and peptides. Co-solvents can provide molecular insight into protein aggregation mechanisms and the chemical nature of potential aggregation inhibitors. Here, we study, through molecular simulations, the hydration and binding free energies of an amphiphilic peptide from the nonamyloid-β component (NAC), a key aggregation-prone domain of α-synuclein, in water and an 8 M aqueous urea solution. Isoleucine, glycine, and serine peptides of the same length are also studied to unravel the role of urea in the hydration and aggregation of hydrophobic and hydrophilic domains. A strong impact of urea in hindering the aggregation of the NAC subdomain is observed. A slightly weaker aggregation inhibition is observed for the Gly and Ser peptides, whereas a much lower aggregation inhibitory activity is found for the Ile peptide, seemingly contrasting with urea’s protein unfolding mechanism. This behavior is shown to derive from a lower profusion of urea next to the hydrophobic side chains and the backbone of the Ile’s peptide in the dimeric form. As a consequence, β-sheets, formed upon aggregation, remain nearly intact. Hydrophilic neighbor groups in the amphiphilic NAC subdomain, however, are shown to anchor enough urea to weaken hydrophobic interactions and disrupt β-sheet structures. Our results indicate that urea’s activity is potentiated in amphiphilic domains and that potential drugs could disrupt hydrophobic β-sheet-rich regions while not binding primarily to hydrophobic amino acids.

## I Introduction

Protein aggregation is implicated in several neurodegenerative diseases, including Alzheimer’s disease and Parkinson’s disease (PD).^1,2^ PD and other synucleinopathies, in particular, have been associated^3−8^ with the formation of cytotoxic oligomers primarily composed of α-synuclein^9^ (α-syn) that accumulate in neuronal inclusions, called Lewy bodies and Lewy neurites. Although the cytotoxicity mechanism remains elusive, these abnormal aggregates, generally referred to as amyloids, are thought to be responsible for the loss of dopaminergic neurons in the substantia nigra pars compacta.^6,7^ α-Syn belongs to the class of natively unfolded proteins, among intrinsically disordered proteins^10^ (IDP), although some compactness, associated with hydrophobic interactions, and transient long-range contacts have been reported from NMR and paramagnetic relaxation enhancement (PRE) experiments.^11−18^ The disordered nature of IDPs is, in general, connected with a relatively low hydrophobic character and a high net charge (*q*_α-syn_ = −9*e*). However, while the hydrophobicity of α-syn is insufficient to induce the formation of stable secondary and tertiary structures, hydrophobic domains seem to be nuclear to aggregation.^19^ Thus, various domains within a largely hydrophobic 35-amino-acid central region, coined nonamyloid-β component (NAC),^20^ comprising residues 61–95, were shown to be pivotal to the aggregation process.

Giasson et al.^21^ reported that the 12-amino-acid region, _71_VTGVTAVAQKTV_82_, of α-syn is necessary and sufficient for its fibrillization. Du et al.^22^ showed that the elimination of the 9-amino-acid sequence _66_VGGAVVTGV_74_ eliminates α-syn fibrillization and cell toxicity. El-Agnaf^23^ concluded that the 74–86 region of NAC was the main binding region responsible for aggregation. Rodriguez et al.^24^ studied the crystal structure of an 11-residue segment comprising residues _68_GAVVTGVTAVA_78_; they coined NACore, indicating its relevance in both the aggregation and cytotoxicity of α-syn. A slightly smaller region, encompassing residues 68–76, had been previously suggested to be pivotal to the cytotoxicity of α-syn.^25^ Another similar-size domain of special interest concerns the domain 72–84 of α-syn, absent in β-synuclein, which, although sharing 78% similarity with α-syn, does not aggregate.^26^

The fact that α-syn can adopt distinct (transient) conformational states can be explored for the development of potential aggregation inhibitors. Bertoncini et al.^15^ and Dedmond et al.^16^ showed that the monomer of α-syn assumes conformations that are stabilized by long-range (tertiary) interactions, involving the C-terminal regions and the NAC, that inhibit aggregation. By contrast, the heterogeneous aggregational nature^27−29^ of α-syn poses serious challenges concerning a comprehensive understanding of the relationship between the aggregation mechanism(s)/kinetics and the onset of idiopathic PD. While several aggregation pathways are possible, a conformational transformation of the natively unfolded protein into a partially folded intermediate with increased β-sheet content, more aggregation-prone, is believed to occur.^30,31^

In the light of the above, a potential inhibitor should either induce or stabilize a less aggregation-prone conformational state of the monomer or repress the growth of nascent aggregates by interacting with specific domain(s) of the natively disordered protein. Nevertheless, the magnitude of the hydration and binding free energy of the above-mentioned domains of α-syn is unknown, limiting a comprehensive understanding of the molecular interactions at play in the early steps of aggregation.

Molecular details on the aggregation mechanism and key protein domains that may serve as potential targets, drugs, or drug leads, in drug design, can be assessed through studies of protein denaturants such as urea. The latter was widely studied concerning protein denaturation^32−39^ and direct and indirect mechanisms put forward. The indirect mechanism posited that protein unfolding was associated with putative structural transformations of water,^40^ which would favor solvation,^41^ weakening hydrophobic interactions,^39^ whereas the direct mechanism^36^ envisaged that interactions of urea with the backbone and side chains are dominant. The direct mechanism is now widely accepted,^42−44^ although the importance of urea, concerning hydrophobic interactions and hydrogen bond (HB) interactions involving backbone and hydrophilic side chains,^37^ remains a matter of debate. Further, while there is experimental^15,45^ and simulation^46−48^ evidence on the role of urea as an antiamyloid agent, its action mechanism and impact on the binding free energy of α-syn or the above-mentioned protein domains remain largely unexplored.

In this work, we studied a C-terminal domain (11-amino-acid peptide) of NAC, composed of the residues _85_AGSIAAATGFV_95_, aiming at getting insight into the magnitude of the hydration and binding free energy in water and an 8 M aqueous urea solution. This domain was chosen as a prototypical region of the NAC, being involved in conformations of the monomer, which could potentially inhibit aggregation.^15^ In particular, the formation of a hydrophobic cluster that comprised the C-terminal domain of NAC (residues 85–95) and the C terminus (residues 110–130), probably mediated by M_116_, V_118_, Y_125_, and M_127_ was identified.^15^ Release of such long interactions was shown to potentiate aggregation of native α-syn.^15,16,18^

In addition, isoleucine (Ile), serine (Ser), and glycine (Gly) peptides of the same length were studied to probe the role of urea in the hydration and aggregation of, respectively, hydrophobic groups, hydrophilic groups, and the backbone.

## II Methods

Molecular dynamics (MD) simulations in the isothermal–isobaric (*N*,*p*,*T*) ensemble of the C-terminal segment of NAC (_85_AGSIAAATGFV_95_), in the zwitterionic form, denoted hereinafter NACterm, were performed in water and an 8 M aqueous urea solution with the program GROMACS.^49^ The peptide and urea were described by the AMBER99sb^50^ force field, whereas water was described by the TIP4P-Ew^51^ model. Further, isoleucine (ILE-11), glycine (GLY-11), and serine (SER-11) peptides composed of 11 amino acids, in the zwitterionic form, were studied; these peptides were chosen to represent a “hydrophobic” (i.e., hydrophobic side chain) peptide, the backbone, as the side chain of Gly is a single H atom, and a hydrophilic peptide. We note that the GLY-11 peptide differs from the backbone of a peptide in that it can be more solvated due to the small size of the Gly side chain. This influences the aggregation of GLY-11 relative to the backbone contribution to aggregation of other peptides where steric effects associated with larger-side-chain amino acids hamper solvation to some extent. Thus, in this sense, GLY-11 can be seen as an ideal model of the backbone for which solvent effects are maximal.

**Figure 1:**
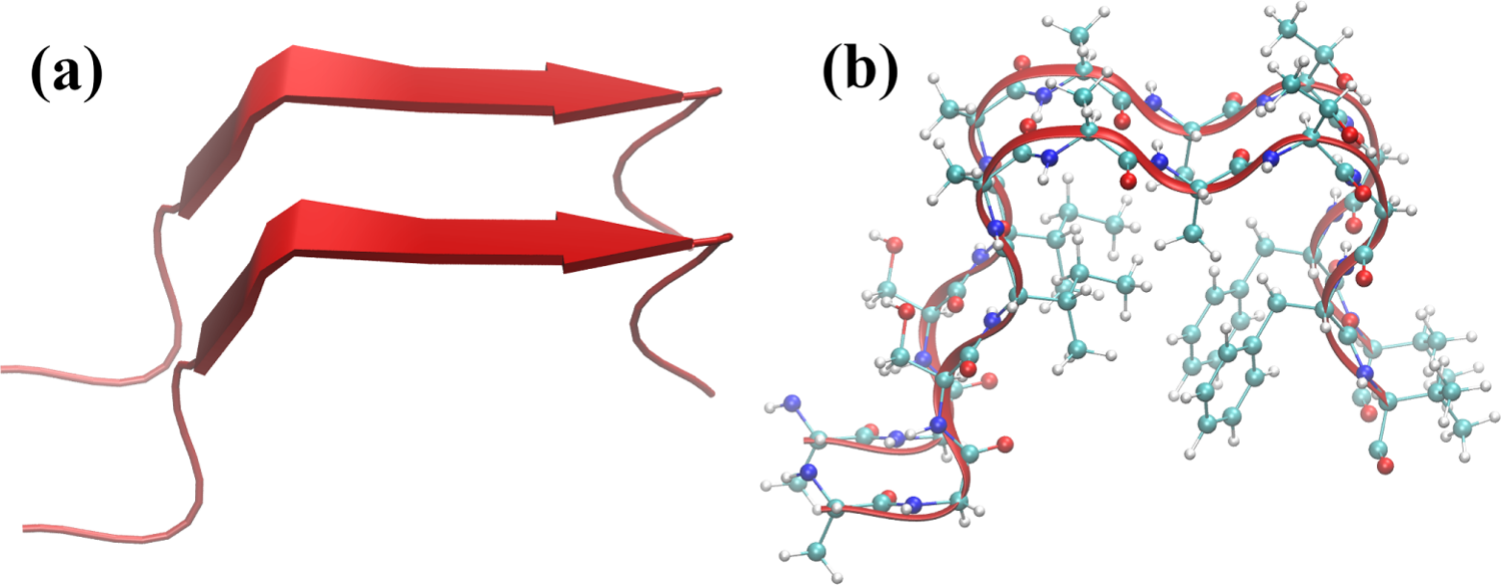
NACterm “dimer”, (A_1_G_1_SIA_2_A_3_A_4_TG_2_FV), extracted from the α-syn experimental protofibril:^52^ (a) cartoon representation showing a β-sheet domain (residues 4 to 8: IA_2_A_3_A_4_T) and (b) ribbons and ball-and-stick representation showing an apparent hydrophobic pocket formed by Ile, Ala (A_4_), and Phe side chains.

MD of the monomers and dimers were performed at 298 K and 0.1 MPa. The starting conformation of the NACterm monomer and dimer (Figure 1) was obtained from the α-syn protofibril reported by Tuttle et al.^52^ (PDB code: 2n0a) from solid-state NMR spectroscopy.

The peptide (A_1_G_1_SIA_2_A_3_A_4_TG_2_FV) “dimer” in the protofibril exhibits a β-sheet region and an apparent hydrophobic cluster involving I, A_4_, and F (see Figure 1).

Molecular dynamics of the monomers were carried out in a cubic box with periodic boundary conditions (PBC), to assess the secondary structure^53^ and the radius of gyration in water and an 8 M aqueous urea solution; the secondary structure was studied with the program DSSP.^53,54^ The AMBER99sb-ILDN^55^ model was also used for the NACterm for comparison purposes; no significant differences were found (see Figure S1a); a similar secondary structure was also found for the α-syn monomer (140 amino acids) with the AMBER99sb force field in TIP4P-EW water, although a lower content of random coil was observed (see Figure S1b).

The trajectories of the peptides were propagated for 1.5 μs in the *NpT* ensemble. The *T* and *p* were controlled with the Nose–Hoover thermostat^56,57^ and the Parrinello–Rahman barostat,^58^ and the equations of motion were solved with the Verlet leap-frog algorithm with a 2 fs time-step. Electrostatic interactions were computed via the particle-mesh Ewald (PME) method.^59^ A cutoff of 1 nm was used for nonbonded van der Waals and for the PME real space electrostatic interactions. Heavy atom–hydrogen covalent bonds were constrained with the LINCS algorithm.^60^

The hydration free energy, Δ*G*_hyd_ (i.e., the excess chemical potential), of the monomers in water and an 8 M aqueous urea solution were calculated through “alchemical” free energy calculations,^61^ with the Bennett acceptance ratio^62^ method. Further details are available elsewhere^63,64^ and in the Supporting Information.

Although the solvation free energy in the aqueous urea solution is not a hydration free energy, the latter designation will be used herein both for water and the aqueous urea solution, for the sake of simplicity. The Δ*G*_hyd_ values of the side-chain analogues^65^ of the amino acids that form the NACterm, with the exception of Gly, were obtained through a similar approach, to validate the peptides and urea force fields, concerning the hydration free energies. The side-chain analogues were built by replacing the C_α_ with an H atom with the same charge as the other H–C_β_, whereas the charge of the C_β_ was changed to turn the side-chain analogue neutral. The remaining force field parameters were kept unchanged.

The binding free energy of the different peptides in water and the 8 M aqueous urea solution was probed through the calculation of the potential of mean force (PMF).^66,67^ The PMFs were calculated through umbrella sampling^68−70^ for a system composed of the respective dimers in a cubic box with PBC, large enough to allow a center of mass (COM) separation of ∼2.7 nm. The reaction coordinate, ξ, was chosen to be the COM distance, ξ = *r*_COM–COM_. The starting configuration of the peptides was the same for the distinct peptides, namely, the position of the peptides A and B in the α-syn protofibril reported by Tuttle et al.^52^ (PDB code: 2n0a); mutations were carried out on the NACterm dimer to generate the remaining peptides.

Following the steepest descent energy minimization and a 20 ns equilibration period in the *NpT* ensemble, the peptides were pulled away with a spring constant of 5000 kJ mol^–1^ nm^–2^ and a pull rate of 0.01 nmps^–1^, through steered MD, to generate initial configurations. A spacing of 0.05 nm was adopted, and the umbrella sampling MD was performed for 200–250 ns after steepest descent energy minimization, a 100 ps equilibration in the *NVT* ensemble, and a 10 ns equilibration in the *NpT* ensemble. The PMFs were obtained through the weighted histogram analysis method^71,72^ (WHAM), and the Bayesian bootstrap method^73^ was used to estimate the PMF errors. The PMFs were corrected for the entropy,^74^ by adding the factor 2*RT*  ln(*r*_COM–COM_), associated with the increasing sampling volume with the COM–COM distance increase. The PMFs were then shifted to have zero free energy at the longest separations.

PMFs of the amino acid analogues Ile/butane and Ser/methanol were also computed through a similar approach; 80–100 ns long umbrella trajectories were carried out to calculate the PMFs.

## III Results and Discussion

**Figure 2:**
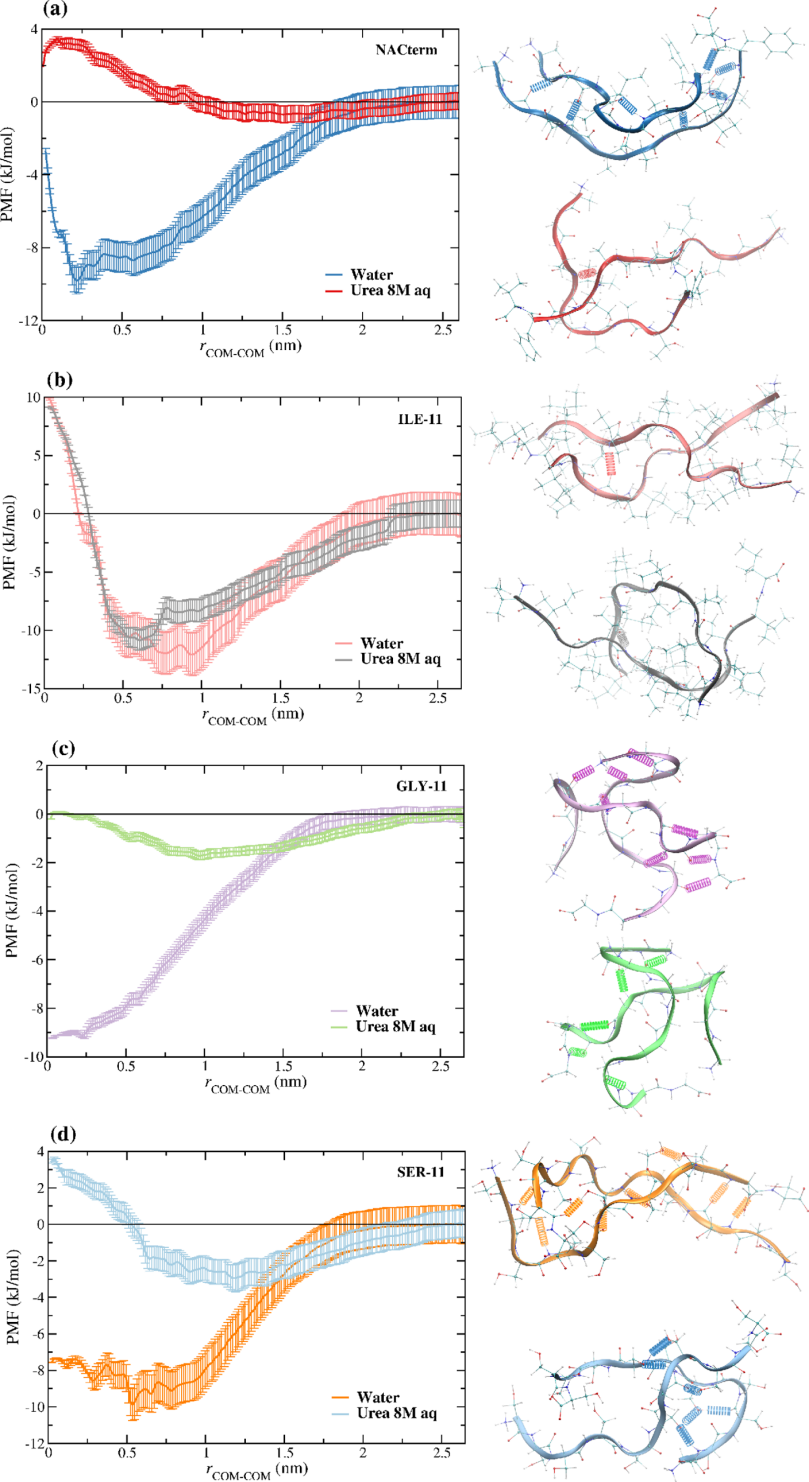
Potential of mean force (PMF) for the (a) NACterm, (b) ILE-11, (c) GLY-11, and (d) SER-11 peptides, in water and an 8 M aqueous urea solution. Umbrella sampling MD snapshots of the respective dimers are shown on the rhs; springs represent intra- and interpeptide backbone and side-chain hydrogen bonds (HBs).

The PMFs for the distinct peptides in water and an 8 M aqueous urea solution are shown in Figure 2. The lowest binding free energy in water is observed for the ILE-11 peptide (∼−12.5 kJ mol^–1^), consistent with the importance of hydrophobic interactions to protein aggregation.^75,76^

The PMF, *W*(ξ), is the average work required to bring two objects from infinite separation to a distance *r*, and it can be written in the form^67,77^
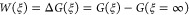
1where *G*(ξ) is the free energy of the system along the reaction coordinate ξ. The COM distance is not, in principle, an optimal reaction coordinate to study the PMF of nonspherical objects, as this can often be located away from the peptide. The choice of suitable reaction coordinates is an intrinsic difficulty of IDPs because of the multiple conformations the proteins can sample. For a small peptide such as NACterm, this is less acute, and previous studies^63^ for a linear alkane of similar length (*n*-dodecane, C_12_H_26_) showed that although the shape of the PMF varies with the choice of either the geometric center or the COM, similar binding free energies are found.

Concerning the effect of urea, a destabilization of the PMF can be observed for the NACterm, with the replacement of a contact minimum by a repulsive state; *W*(ξ) increases by Δ*W*^W → U^(ξ_min_) ∼130% at the equilibrium distance, ξ_min_, upon the transference of the peptides from water to aqueous urea solution. A destabilization is also found for the GLY-11 and SER-11 peptides, with the appearance of shallow minima at longer distances, resembling a solvent-separated state. The most remarkable feature of Figure 2 is, however, the much lower aggregation inhibitory activity of urea on the ILE-11 peptide dimer; Δ*W*^W → U^(ξ_min_) ∼32%. While unforeseen, in view of urea’s induced protein unfolding mechanism,^38^ this behavior is consistent with recent results^63^ for OPLS-aa *n*-dodecane in TIP4P/2005 water, which showed that urea slightly enhances aggregation in spite of favoring hydration. However, the “mutation” of some CH_2_ groups into charged groups allowed inverting this enhanced aggregation propensity.^63^

**Figure 3:**
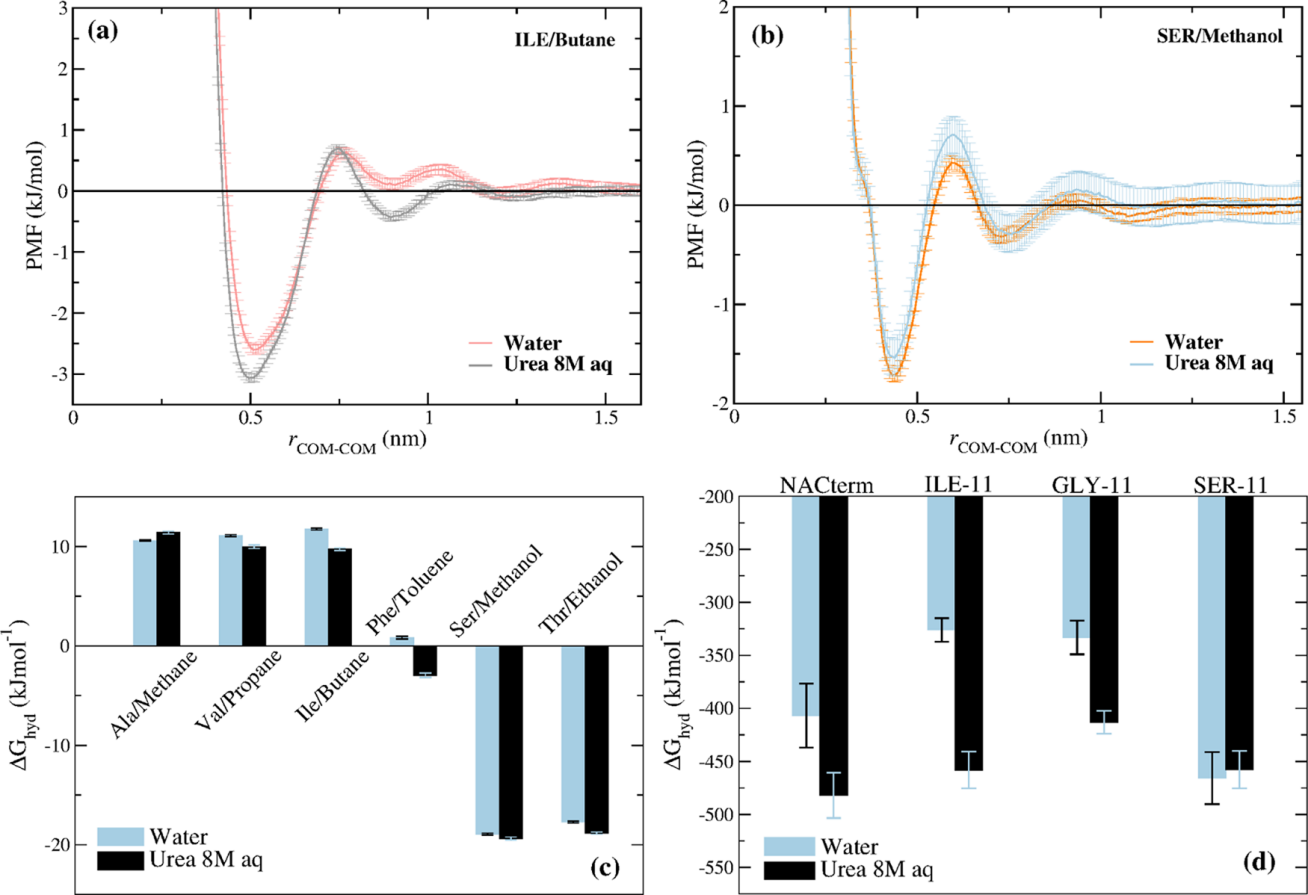
PMF for the (a) Ile/butane and (b) Ser/methanol analogues and hydration free energy in water and an 8 M aqueous urea solution for the (c) NACterm amino acids’ side-chain analogues and (d) the peptides.

Figure 3a,b shows the PMF of the Ile/butane and Ser/methanol analogues, confirming that urea induces a slight stabilization of the PMF of the former, whereas a minor destabilization is observed in the latter. Notice that unlike for the peptides a desolvation barrier can be seen, separating a contact minimum from a solvent-separated minimum. Urea stabilizes the solvent-separated minimum in the Ile/Butane analogue, but no increase in the desolvation barrier is observed, whereas in Ser/Methanol, the solvent-separated minimum remains unchanged but the desolvation barrier is enhanced.

To understand whether a direct or inverse relationship is observed for the peptides, concerning the hydration and aggregation propensity, the hydration free energy was calculated. Further, Δ*G*_hyd_ of the side-chain analogues of each amino acid in the NACterm was computed. These results are displayed in Figure 3c,d. A good agreement with experimental data is found for the Δ*G*_hyd_ of the side-chain analogues, with the exception of Phe/Toluene for which a small positive value is found, opposite to the experimental value (see Table 1). Table 1 also shows that the urea model accurately describes the positive experimental^41^ free energy of transfer from water to aqueous urea solution (ΔΔ*G*_hyd_ > 0) of methane and ΔΔ*G*_hyd_ < 0 for alkanes larger than ethane. This result was recently shown by our group^63^ to be accurately reproduced with the OPLS-aa force field for urea but not by a force field^78^ for urea that provides a more accurate description of urea–water mixtures, when combined with the alkanes’ OPLS-aa force field.

**Table 1:** Hydration Free Energy of the Amino Acid Side-Chain Analogues that form NACterm, with the Exception of Gly, in Water and an 8 M Aqueous Urea Solution

aa/analogue	MD^a^ water Δ*G*_hyd_ (kJ mol^–1^)	exp.^b^ water Δ*G*_hyd_ (kJ mol^–1^)	MD urea 8 M aqueous Δ*G*_hyd_ (kJ mol^–1^)	MD ΔΔ*G*_hyd_^c^ (kJ mol^–1^)
Ala/methane	+10.6 ± 0.07	+8.4	11.4 ± 0.1	+0.8 ± 0.1^d^
Val/*n*-propane	+11.1 ± 0.1	+8.2	10.0 ± 0.2	–1.1 ± 0.2
Ile/*n*-butane	+11.8 ± 0.09	+8.7	9.7 ± 0.1	–2.0 ± 0.1
Phe/toluene	+0.8 ± 0.1	–3.7	–2.9(5) ± 0.2	–4.0 ± 0.2
Ser/methanol	–18.9 ± 0.1	–21.3	–19.4 ± 0.2	–0.45 ± 0.2
Thr/ethanol	–17.7 ± 0.1	–21.0	–18.8 ± 0.1	–1.1 ± 0.1

a The hydration free energies were estimated from two independent calculations; the errors were estimated through error propagation analysis.

b Experimental values: ref (80).^80^

c Water to aqueous urea solution transfer free energy; ΔΔ*G*_hyd_ = Δ*G*_solv_(U) – Δ*G*_hyd_(W); U = urea; W = water.

d The experimental value^41^ of ΔΔ*G* for methane is 0.8 kJ mol^–1^ when converted to the Ben–Naim standard state;^81,82^ the values of Δ*G*_hyd_(W) and Δ*G*_hyd_(U), respectively, obtained with the OPLS-aa force field in TIP4P/2005 water are^63^ 9.4 ± 0.1 and 10.5 ± 0.1.

A significant effect of urea can be seen for Phe/Toluene (ΔΔ*G*_hyd_ = −4.0 ± 0.2), indicating that urea has a pronounced influence on the solvation of aromatic rings. In this sense, a Phe-11 peptide also represents a relevant model to probe the effect of urea in peptide aggregation. However, aromatic rings have both hydrophobic (CH groups) and hydrophilic regions (π-electrons modeled by the excess negative charge in some C atoms), forming HBs as proton acceptors, thus turning the disentanglement of hydrophobic and hydrophilic effects more difficult. In addition, α-syn has only four tyrosine (Tyr39, Tyr125, Tyr133, Tyr136), two phenylalanine (Phe4, Phe94), and no tryptophan amino acids, of which only Phe94 is in the NAC segment. That suggests that aromatic rings are not key players in the α-syn aggregation or the urea-induced disaggregation.

The Δ*G*_hyd_ values in neat water for the amino acid analogues are also in very good agreement with a previous simulation study,^65^ although with the TIP3P water model. The good agreement with experimental hydration free energies and the fact that the AMBER99sb/TIP4P-Ew models can reproduce the structure^79^ of the Aβ_42_ peptide, implicated in Alzheimer’s disease, supported our choice of this force field.

Concerning the peptides, Figure 3d shows that urea favors the solvation of the NACterm, ILE-11, and GLY-11, whereas, for SER-11, urea seems to play a minor role. The most marked decrease of Δ*G*_hyd_ is found for ILE-11, challenging the common idea that a solvation enhancement reduces the aggregation propensity of the peptide.^83^

The decrease of the Δ*G*_hyd_ of hydrophobic solutes in aqueous urea solutions is entropic^41^ and is thought to be associated with a water depletion next to the solute, restoring water molecules’ rotational and translational freedom, as these are replaced by urea.^63,84^ Although solute–solvent interactions are favorable, these are overwhelmed by urea–water and urea–urea interactions,^63^ resulting in a positive hydration enthalpy.^41,63^ Although ILE-11 is amphiphilic, because of the backbone, comparison with GLY-11 indicates that urea should favor the solvation of the side chains (*n*-butane), in keeping with the negative transfer free energy ΔΔ*G*_hyd_ < 0 of the Ile/butane analogue (see Table 1).

The reason for urea to significantly favor solvation, while not reducing the aggregation propensity of ILE-11, should then be connected with differences between solvation when in the monomeric and dimeric forms. The hypothesis exploited herein foresees that whereas the urea-induced dehydration next to a hydrophobic group favors solvation (ΔΔ*G*_hyd_ < 0), a similar dehydration would favor aggregation unless urea’s profusion is enough to form a “surfactant” layer^43^ that prevents the hydrophobic collapse and the formation of interpeptide backbone hydrogen bonds (HBs). That is to say, unless enough urea remains in the solvation layers of the hydrophobic groups upon the peptides’ approximation, neither hydrophobic interactions nor backbone HBs may be completely disrupted.

**Figure 4:**
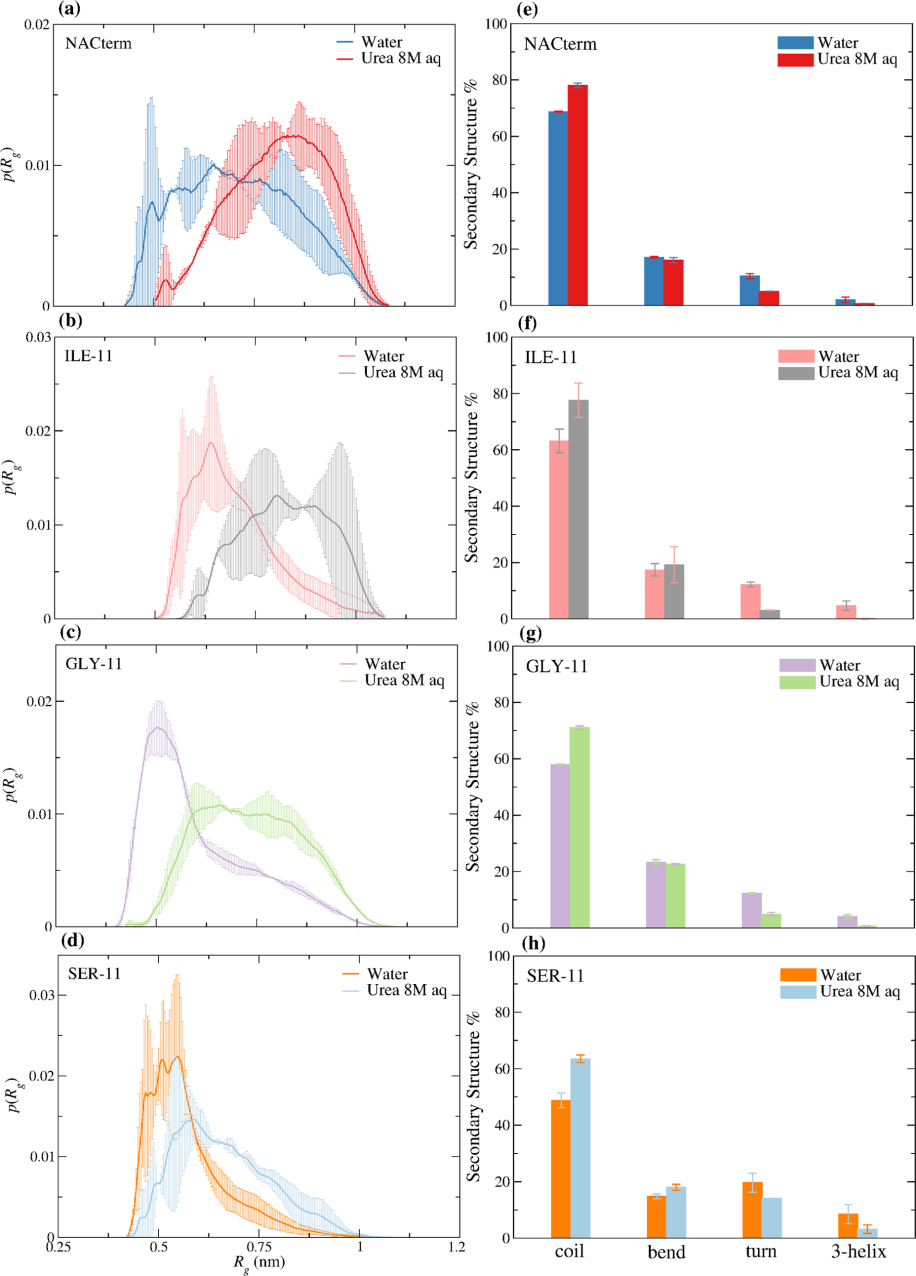
(a–d) Radius of gyration, *R*_*g*_, distributions for the peptides (monomers) and (e–h) secondary structure for the peptides (monomers) in water and 8 M aqueous urea solution.

Before we discuss solvation, however, we analyze the structural differences between the peptides in water and the aqueous urea solution. The structure was probed by calculating the radius of gyration, *R*_*g*_, and the secondary structure of the peptides. *R*_*g*_ provides a measure of the peptide compactness, with larger values corresponding to more extended conformations. These results are shown in Figure 4a–d for the monomers. The most compact average conformation in water is found for GLY-11, whereas the least compact is observed for ILE-11, possibly because of steric effects. Urea induces a less compact conformation for the four peptides, similar to the effect observed in hydrophobic polymers^85^ and globular proteins.^43^

Figure 4e–h shows the main secondary structures of the monomers; an energetic HB criterion (*E*_HB_ < −0.5 kcal mol^–1^ = −2.09 kJ mol^–1^) is used to define backbone NH···O HBs in the DSSP^53^ analysis.

The peptides exhibit neither α-helix nor β-sheet structures, with the highest percentage of random coil observed for the NACterm and ILE-11. Urea induces an increase in the percentage of random coil for every peptide and the decrease of turns (single HB helix segment^53^) and 3_10_-helices, nearly absent in the NACterm, even in water.

**Figure 5:**
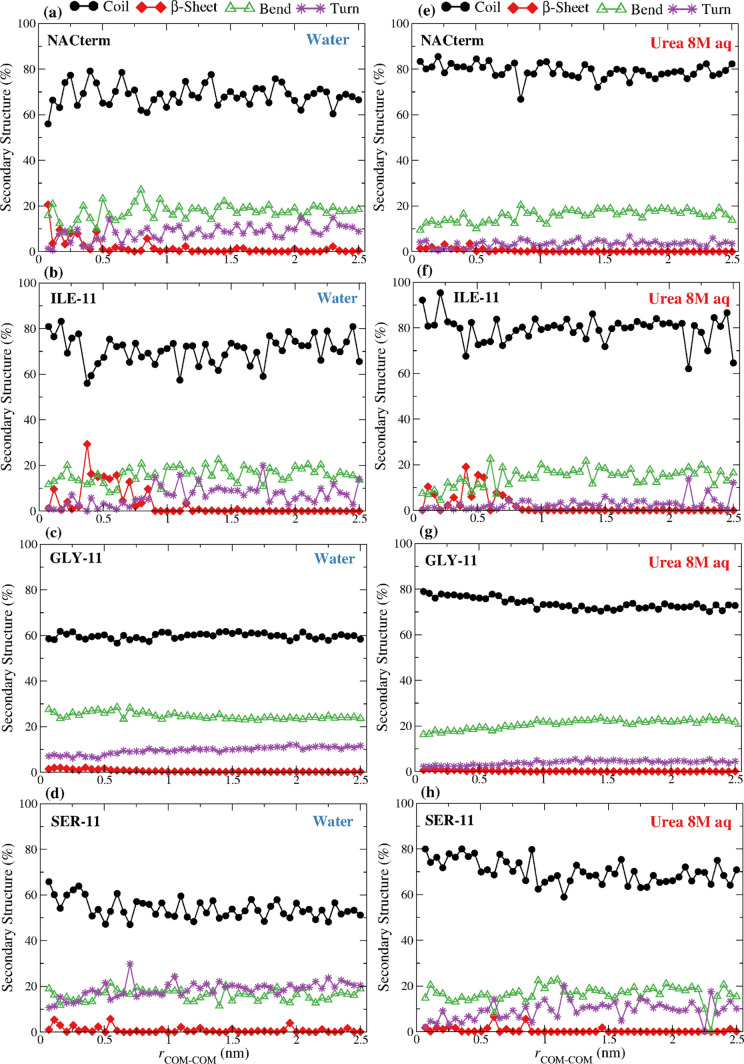
Secondary structure of the peptides (dimers), calculated from the umbrella sampling trajectories in (a–d) water and an (e–h) 8 M aqueous urea solution; lines are only a guide to the eye.

Figure 5a–d displays the secondary structure of the dimers assessed from the umbrella sampling trajectories in water at every COM–COM distance. These show the appearance of β-sheet structures in the NACterm and ILE-11, upon aggregation, consistent with the β-sheet structures that characterize α-syn and other IDPs transient oligomers. In α-syn, these structures appear in the region of the NAC, a largely hydrophobic domain. Thus, it is interesting to observe that the β-sheet structures appear primarily in ILE-11 but are nearly inexistent in GLY-11 and SER-11. Furthermore, it can be seen that the distances at which the β-sheets appear nearly overlap with the respective minima of the PMFs, indicating that this is a structural hallmark of the dimer in the equilibrium state.

Similar plots for the dimers Figure 5e–h in the aqueous urea solution show the disruption of these β-sheet structures and the increase in the random coil content, similar to the monomers, with the exception of ILE-11. Thus, urea is unable to disrupt these structures in ILE-11, suggesting that interpeptide backbone HBs survive upon urea’s intrusion into the solvation spheres.

To gain further insight into the dimers’ structural transformations upon transference from water to the aqueous urea solution, interpeptide backbone carbonyl–amino (O–N) neighbor maps were computed from the umbrella sampling trajectories at every COM–COM distance. These were built by calculating the backbone carbonyl–amino (O–N) interpeptide radial distribution functions (RDFs). Every pair at a distance *r* ≤ 3.5 Å was considered to be a neighbor with the potential to engage in an interpeptide HB. This is nearly the distance of the first minimum of the O–N RDF at most interpeptide distances where neighbors are found and the distance commonly used in geometric HB definitions of water.

**Figure 6:**
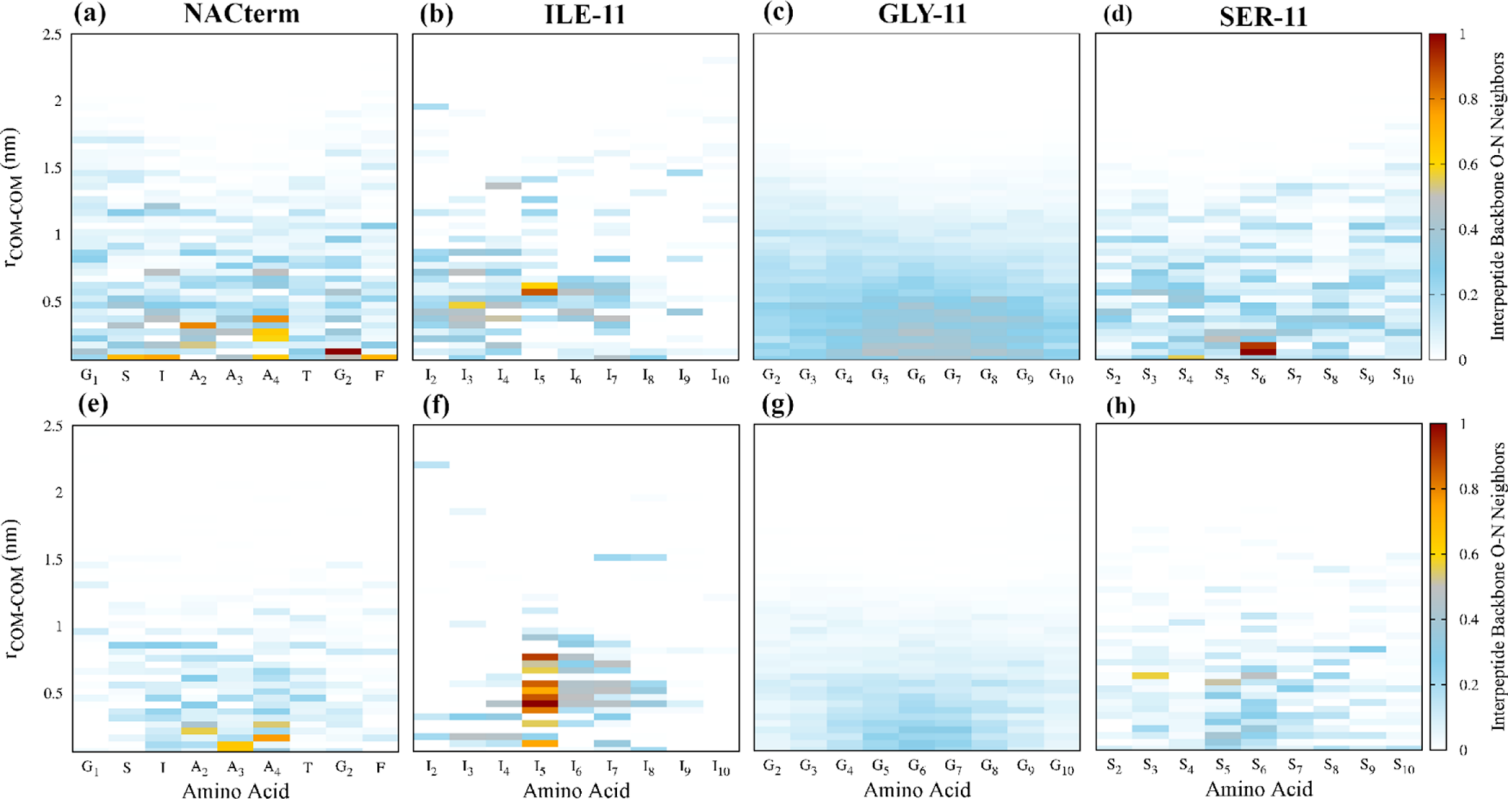
Interpeptide backbone carbonyl–amino (O–N) neighbor maps computed from umbrella sampling trajectories at every COM–COM distance in (a–d) water and (e–h) an 8 M aqueous urea solution. Each number of neighbors is averaged over the same amino acid in the two peptides.

Figure 6 shows a general decrease in the number of interpeptide backbone O–N contacts upon the transference from water to the aqueous urea solution. The lowest and highest number of interpeptide backbone neighbors are found, respectively, for ILE-11 and GLY-11. This shows that hydrophobic interactions, and not interpeptide backbone HBs, are responsible for the lower binding free energy of ILE-11 (see Figure 2). However, in the aqueous urea solution, there is a slight strengthening of the number of neighbors in the central amino acids (I_5_–I_7_) in ILE-11, not observed for the other peptides. This confirms that urea does not significantly destabilize the interpeptide backbone HBs involved in the formation of the β-sheet structures, suggesting a milder penetration of the denaturant. We anticipate that this is indeed the reason and that a significantly lower profusion of urea is found next to the side chains and backbone of ILE-11.

**Figure 7:**
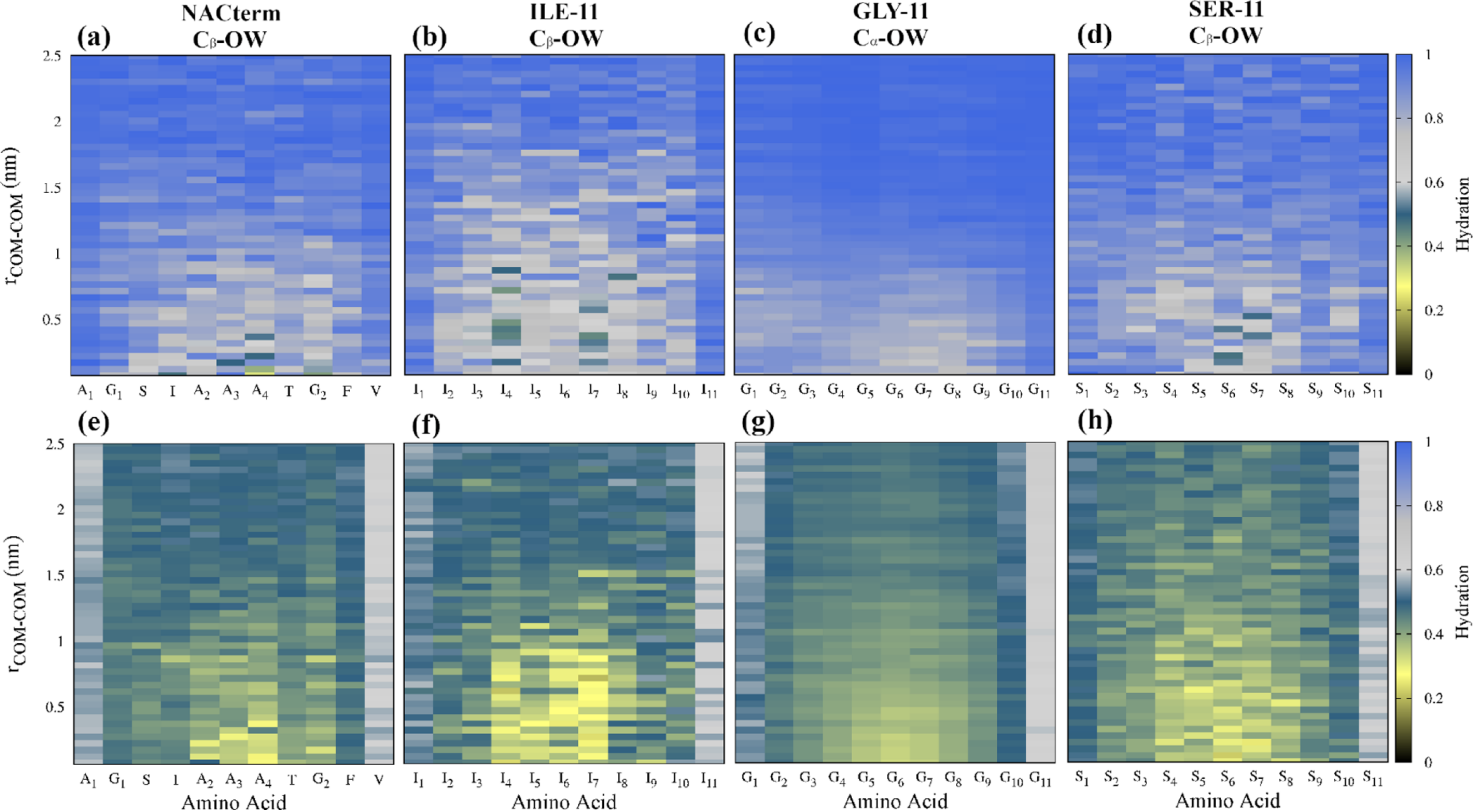
Hydration maps for the distinct peptides in (a–d) water and (e–h) an 8 M aqueous urea solution computed from umbrella sampling trajectories at every COM–COM distance. Hydration is defined by the number of water molecules in the first hydration sphere of the C_β_ (C_α_ was used for Gly) of the amino acids; the hydration spheres were defined by the first minimum of the respective RDFs. Hydration numbers in water and the aqueous urea solution were normalized by the maximum hydration numbers of each amino acid in water. Note that the N-terminus (−NH_3_^+^; amino acid 1) and the C terminus (−COO^–^; amino acid 11) are significantly less dehydrated than the remaining residues.

To probe the hydration level next to the peptides, hydration maps were calculated from the umbrella sampling trajectories. These were built by calculating the amino acids C_β_–OW (C_α_–OW for glycine) (see Figure 7) and the backbone O–OW (Figure S2) and N–OW (Figure S3) coordination numbers (CNs), along the PMF reaction path, where OW is the water molecules’ oxygen atom

The CNs in water and urea were both normalized by the maximum CNs for each amino acid in water
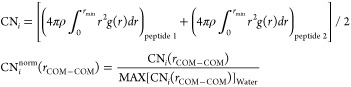
2where CN*_i_* is the CN of amino acid *i* averaged over the two peptides, *g*(*r*) is the RDF, *r*_min_ is the first minimum of the respective RDF, and CN_*i*_^norm^ is the normalized CN for amino acid *i*.

Figure 7a–d shows a clear hydration/dehydration transition as the peptides approach. An even larger dehydration is observed next to the backbone O atoms and especially the N atoms (see Figures S2 and S3). The most prominent dehydration, upon aggregation, is found for ILE-11 in consonance with the expected dewetting and hydrophobic collapse, the hallmarks of hydrophobic aggregation.^86,87^ For the NACterm, the alanine residues (A2, A3, and A4) including the respective backbone O and N atoms, and T and G_2_, to a less extent, are significantly more dehydrated than the remaining amino acids. A more pronounced dehydration is naturally expected in the central region, where interpeptide backbone contacts are also maximized upon association (see Figure 6).

In the aqueous urea solution (Figure 7e–h), most residues display a dehydration of ∼50% at large separations and >65% when the peptides are in contact (<1 nm). This is consistent with urea’s ability to displace water molecules next to both hydrophobic and hydrophilic groups because of a more favorable interaction of these groups with urea than with water.^43^ However, the most striking dehydration is observed for ILE-11, which could suggest a larger profusion of urea.

**Figure 8:**
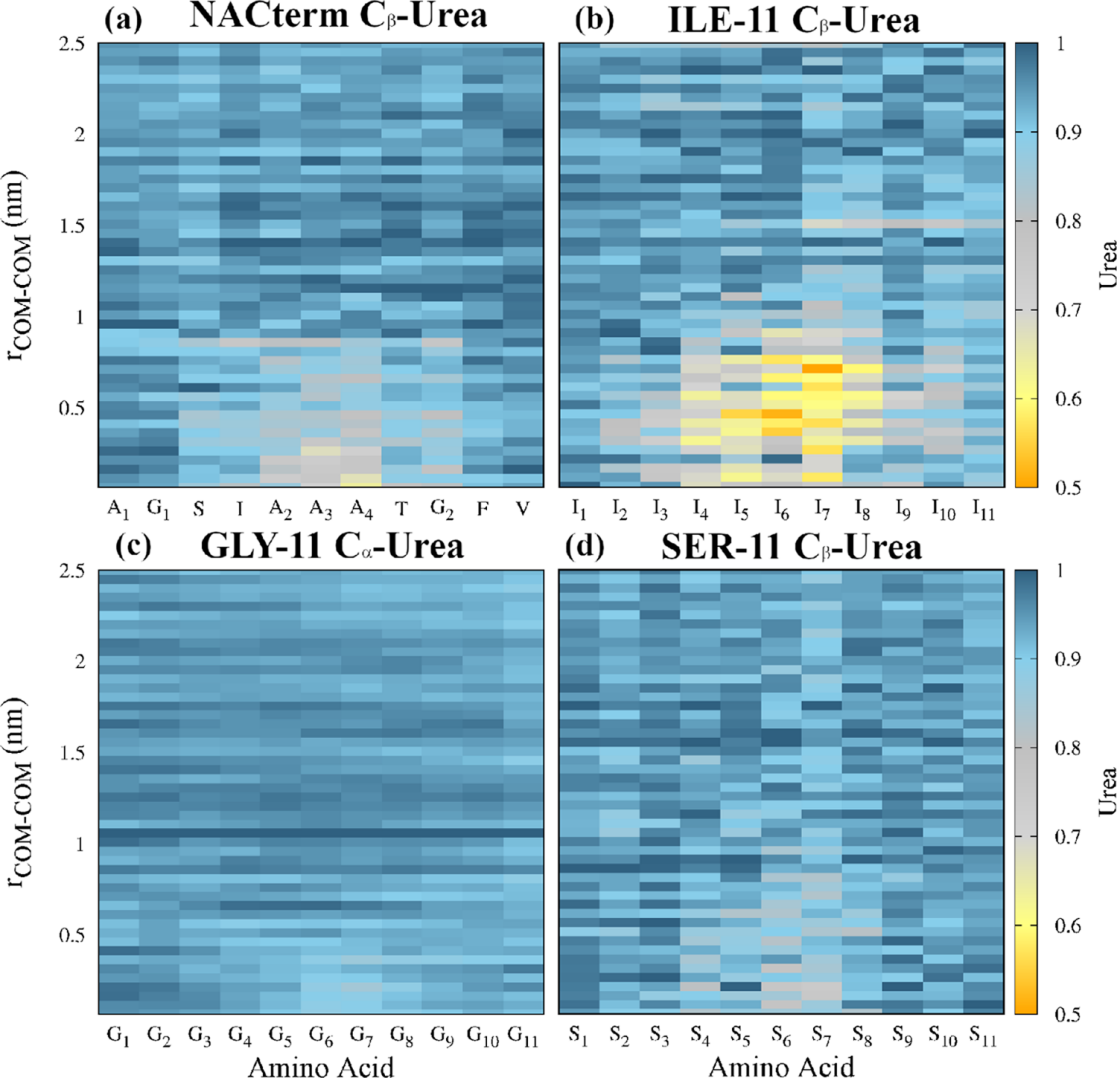
Urea solvation maps of the peptides computed from umbrella sampling trajectories at every COM–COM distance. Solvation is defined by the number of urea molecules in the first urea coordination sphere of the C_β_ (C_α_ was used for Gly), normalized by the respective maximum coordination number found for each amino acid.

Urea solvation maps, however, contradict this expectation and show a lower intrusion of urea in the interpeptide region in ILE-11 (Figure 8), specially marked near the backbone atoms (Figures S4 and S5).

A larger urea depletion is also observed next to the central Ala amino acids in NACterm, although less pronounced.

Thus, depletion of both water and urea is found near long hydrophobic regions, explaining the poor aggregation inhibitory effect of urea observed for ILE-11.

Nonetheless, the larger impact of urea in the aggregation of the NACterm than in GLY-11 and SER-11 indicates that the role of urea in blocking hydrophobic interactions is especially important. However, that depends on the retention of enough urea around the peptides. This is achieved in NACterm through the interaction of hydrophilic groups with urea. Thus, for instance, Ile in the NACterm is well solvated by urea because it shares the solvation layer with a neighbor Ser.

Finally, while the binding entropy and enthalpy cannot be assessed from our results alone, an interesting aspect concerns the role of urea in the intrapeptide and interpeptide interactions (enthalpic). We found a major increase in the interpeptide potential energy (Figure S6) for every peptide, except ILE-11, for which the potential energy profile shows only a moderate increase. A similar behavior is found for *U*_intra_(*r*) (Figure S7). Thus, urea increases the binding enthalpy via interpeptide and intrapeptide interactions favoring the disaggregated state. The urea-induced release of water molecules around the peptides to the bulk is also expected to favor disaggregation through both entropy and enthalpy, whereas water–urea and urea–urea may exert the opposite effect as these were found to disfavor the solvation of hydrophobic solutes.^63^

## IV Conclusions

Urea is routinely used as a denaturant in protein unfolding/refolding and aggregation studies in vitro. While a molecular picture of urea’s protein unfolding mechanism emerged in recent years, less is known concerning peptide and protein aggregation, implicated in several cell and neurodegenerative diseases. Here, we studied the solvation and aggregation of NACterm, an amphiphilic peptide from NAC, a key aggregation-prone domain of α-syn, implicated in several synucleinopathies. Furthermore, “hydrophobic”, backbone, and hydrophilic peptide models were studied. Our results indicate that urea’s role in the aggregation of long hydrophobic domains is limited by a poor profusion near the side chains and the backbone, upon aggregation. Thus, while urea’s profusion around the monomer is enough to favor solvation, in the dimer, this is insufficient to compensate for dehydration, and, therefore, overcome hydrophobic interactions.

These results demonstrate that the effect of urea on protein aggregation (and denaturation) is amplified in amphiphilic domains, as hydrophilic groups anchor enough urea molecules to inhibit hydrophobic interactions as seen for the NACterm, and in protein denaturation.^38^ This explains the seemingly paradoxical result that a significant solvation enhancement does not translate into a significant aggregation propensity inhibition, a result consistent with the inverse relationship between solvation and aggregation in alkanes larger than ethane.^63^

In spite of the heterogeneous nature of α-syn oligomers, there is a common acceptance that any drug, either a small molecule or a peptide-based drug,^88^ that can shield the NAC region^15,16^ is of potential therapeutic interest. In this respect, small molecules are generally less specific and potent than peptide-based antiamyloid drugs because the main target domains are hydrophobic, and, therefore, molecules with large hydrophobic surface areas are desired. This rationale is used, for instance, in designing peptide-based drugs.^23^ Our results, however, indicate that drugs without a primary binding affinity toward hydrophobic domains can still have antiamyloid activity by interacting with neighbor hydrophilic groups in the NAC region. This may, thus, be explored in the design of small molecules and/or macrocyclic peptides with antiamyloid activity. A limitation of this study, however, concerns the size of the peptide studied, in that, despite the expected importance of the NACterm to the aggregation of α-syn, it cannot reproduce transient structural conformations associated with intramolecular interactions between the NAC and the terminal regions of the α-syn. These have an impact on urea and/or drug profusion, which, in turn, also impact the structure of α-syn.

## Supporting Information Available

Hydration free energy methodological details, secondary structure of the NACterm and α-syn, hydration maps of the backbone O and N atoms, urea solvation maps of the backbone O and N atoms, and inter- and intrapeptide potential energy profiles (PDF)
