# Supplementary material for: Aggregation of a Parkinson’s Disease-Related Peptide: When Does Urea Weaken Hydrophobic Interactions?
Source: ACS Chem Neurosci. 2022 May 26;13(12):1769–81. doi: 10.1021/acschemneuro.2c00169 (PMC9775218; doi:10.1021/acschemneuro.2c00169)
Supplement: Supplementary file 1 — cn2c00169_si_001.pdf [file cn2c00169_si_001.pdf]

Aggregation of a Parkinson's Disease-Related Peptide: When does Urea Weaken Hydrophobic Interactions?

Supporting Information

Galamba, N.[a,*]

[a] Biosystems and Integrative Sciences Institute, Faculty of Sciences of the University of Lisbon, C8, Campo Grande, 1749-016 Lisbon, Portugal.

*Corresponding author. Electronic mail: njgalamba@fc.ul.pt

Method Details

A. Hydration Free Energies

The hydration free energies were obtained through "alchemical" free energy calculations[1] with the Bennett acceptance ratio (BAR)[2] method. This method involves the perturbation of the system based on the definition of a parameter, λ, taking values in the interval [0,1], allowing connecting the end states of interest, *A* (λ=1) and *B* (λ=0), defined by the Hamiltonians $\mathbf{H}_A(r,p;\lambda)$ and $\mathbf{H}_B(r,p;\lambda)$. The transition from state *A* (solution) to state *B* (solvent), is performed by a number (N_λ) of different values of λ, corresponding to non-physical states. The ΔG_{hyd} calculated in this work are for the transfer of a solute from a fixed position in the gas phase to a fixed position in water, following the Ben-Naim and Marcus[3] standard, as opposed to the transfer from a non-polar environment to water.

A decoupling approach was used with $N_\lambda = 20$, connecting the states *A* and *B*. For the Coulombic interactions decoupling, a $\Delta\lambda = 0.25$ was adopted [0.0 0.25 0.5 0.75 1.0], whereas for the van der Waals interactions $\Delta\lambda = 0.05$ and 0.1 were used [0.0 0.05 0.1 0.15 0.2 0.25 0.3 0.35 0.4 0.45 0.5 0.55 0.6 0.65 0.7 0.75 0.8 0.85 0.9 0.95 1.0]. For the peptides, the influence of N_λ was assessed by performing additional calculations for $N_\lambda = 32$ and distinct sets of λ values, decreasing the $\Delta\lambda$ for the Coulomb and van der Waals interactions. The ΔG_{hyd} were estimated by averaging over two and five alchemical simulations, starting from different initial velocities, for the amino acid side chain analogs and the peptides, respectively. Although large standard deviations were found for the peptides no apparent dependence of N_λ was observed. Langevin stochastic MD[4] were carried out and a soft-core potential was used for the Lennard-Jones and electrostatic interactions to avoid numerical singularities at terminal λ values, with α = 0.5, σ = 0.3, and a soft-core power of 1[1,5–8]. The simulations for each λ consisted of a steepest descent energy minimization step, followed by a 0.5 ns Langevin *NVT* simulation, and a 1 ns Langevin simulation in the *NpT* ensemble, using the Parrinello-Rahman barostat[9]. The hydration free energies were computed from independent Langevin *NpT* simulations, 10 ns long, for each λ. A more conservative time-step of 1 fs was used to avoid intramolecular instabilities throughout the decoupling simulations, for the urea solutions, whereas a time-step of 2 fs was used for the neat water solutions.

Figures

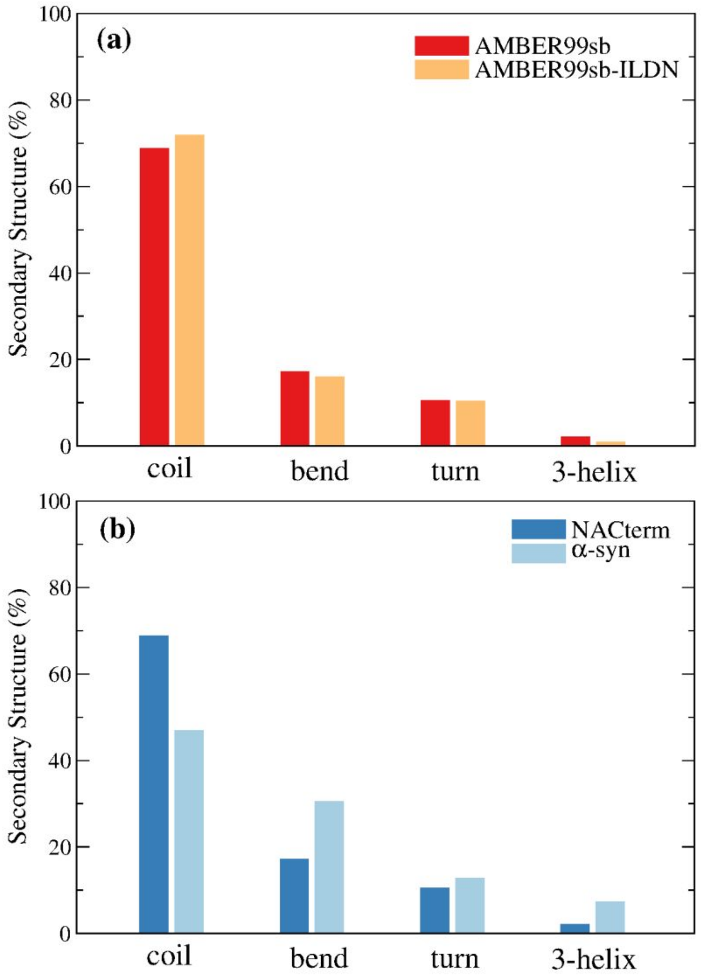

Figure S1 – **(a)** Secondary structure of the NACterm peptide in TIP4P-Ew water calculated with the AMBER99sb and the AMBER99sb-ILDN force fields; **(b)** Secondary structure of the NACterm peptide and of the α-syn protein in TIP4P-Ew water calculated with the AMBER99sb force field. The structure of α-syn was computed through analysis of a 400 ns trajectory in the isothermal-isobaric ensemble at 298 K and 0.1 MPa.

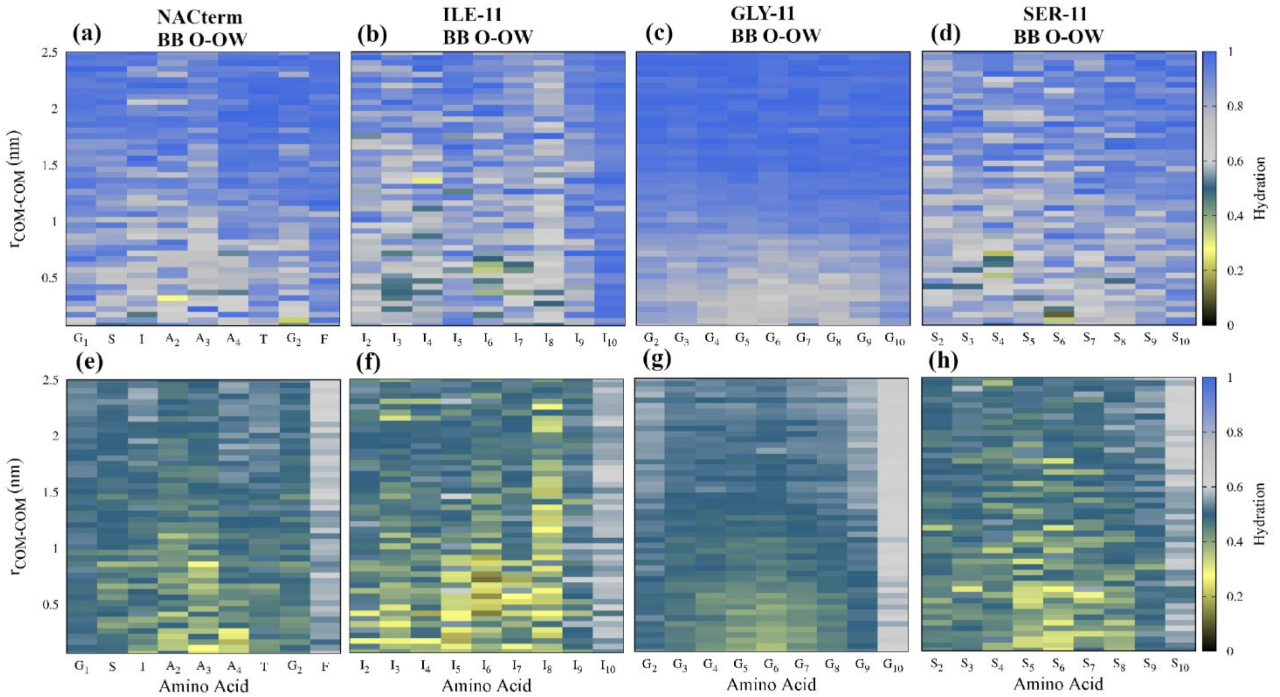

Figure S2 – Backbone carbonyl hydration maps for the distinct peptides in **(a-d)** water and **(e-h)** an 8M aqueous urea solution, computed from umbrella sampling trajectories at every COM-COM distance. Hydration is defined by the number of water molecules in the first hydration sphere of the backbone O (BB O-OW) of the amino acids 2-10. Hydration in water and in the aqueous urea solution were normalized by the maximum hydration number of each amino acid in water.

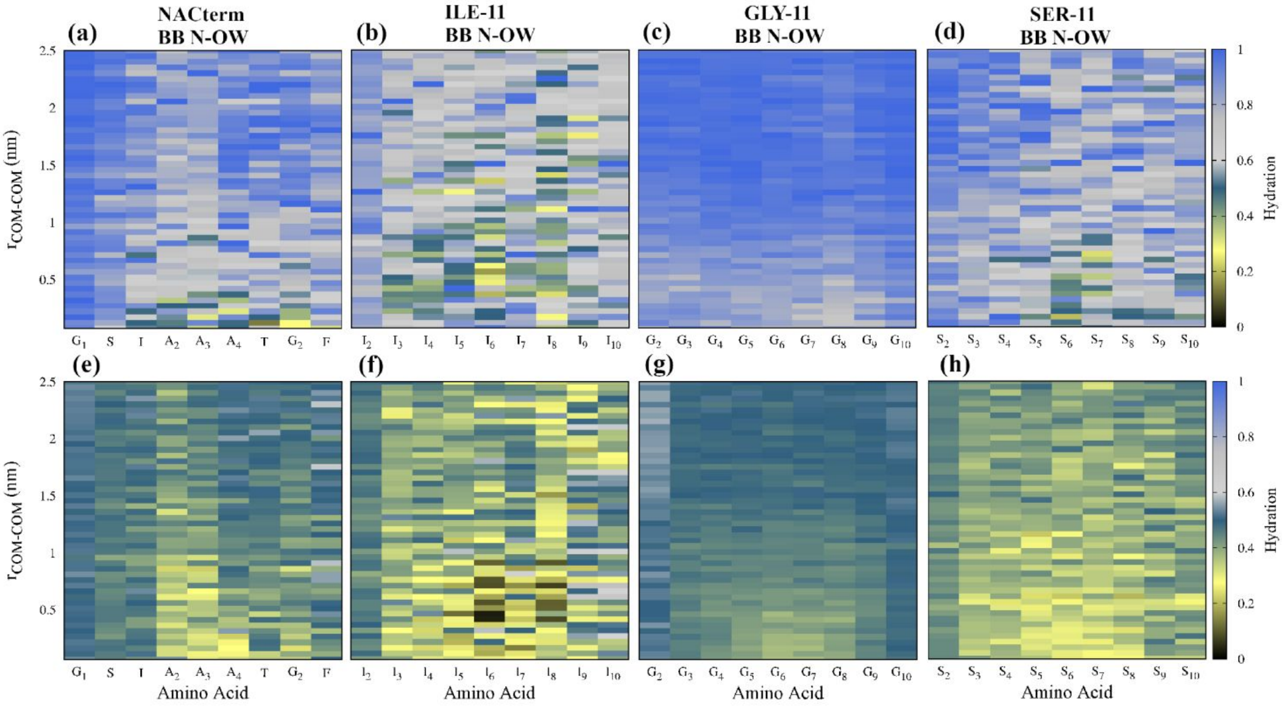

Figure S3 - Backbone amino hydration maps for the distinct peptides in **(a-d)** water and **(e-h)** an 8M aqueous urea solution, computed from umbrella sampling trajectories at every COM-COM distance. Hydration is defined by the number of water molecules in the first hydration sphere of the backbone N (BB N-OW) of the amino acids 2-10. Hydration in water and in the aqueous urea solution were normalized by the maximum hydration number of each amino acid in water.

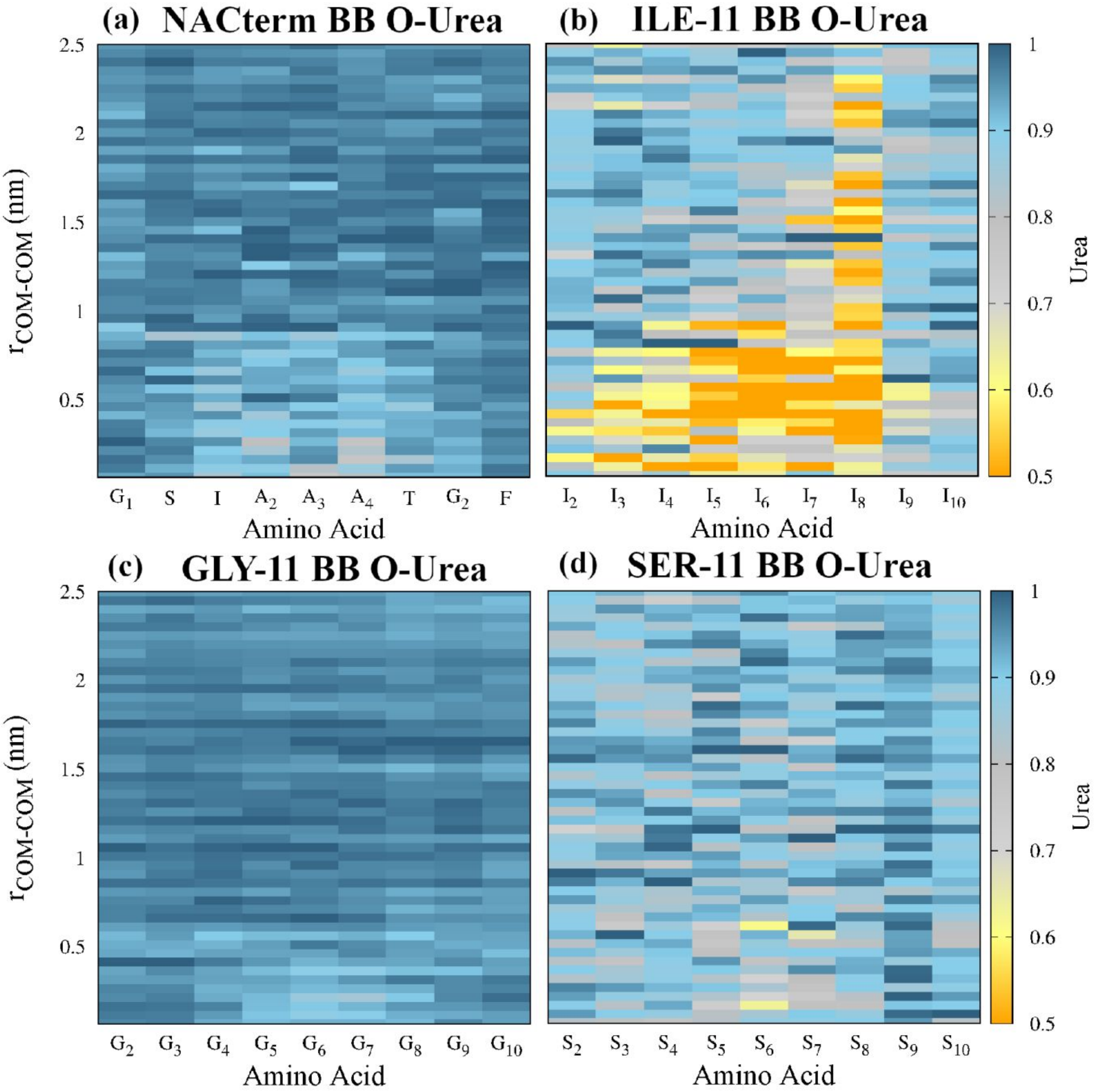

Figure S4 - Urea solvation maps of the peptides computed from umbrella sampling trajectories at every COM-COM distance. Solvation is defined by the number of urea molecules in the first urea coordination sphere of the backbone carbonyl (O atom), normalized by the respective maximum coordination numbers found for each amino acid.

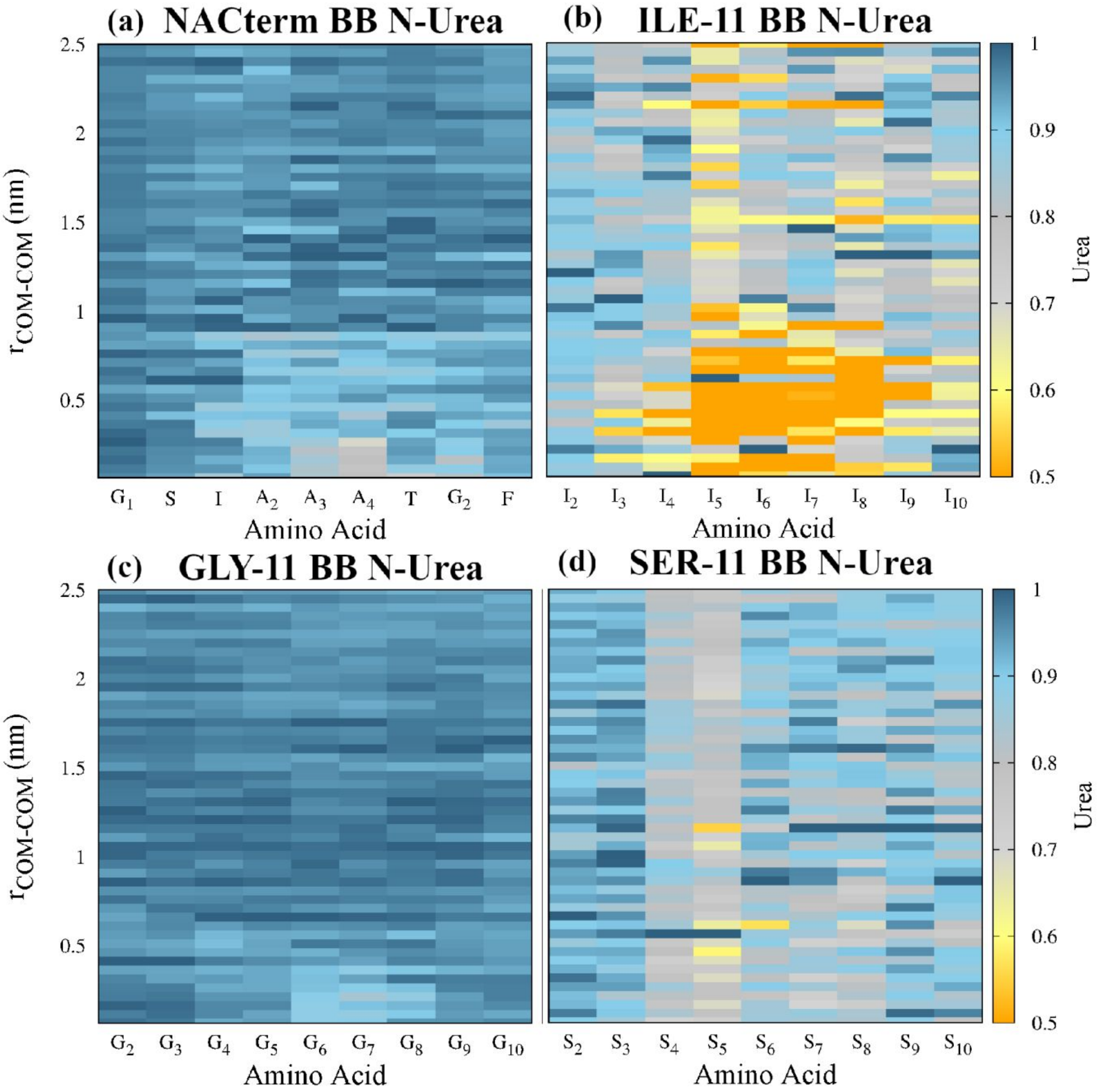

Figure S5 - Urea solvation maps of the peptides computed from umbrella sampling trajectories at every COM-COM distance. Solvation is defined by the number of urea molecules in the first urea coordination sphere of the backbone amino group (N atom), normalized by the respective maximum coordination numbers found for each amino acid.

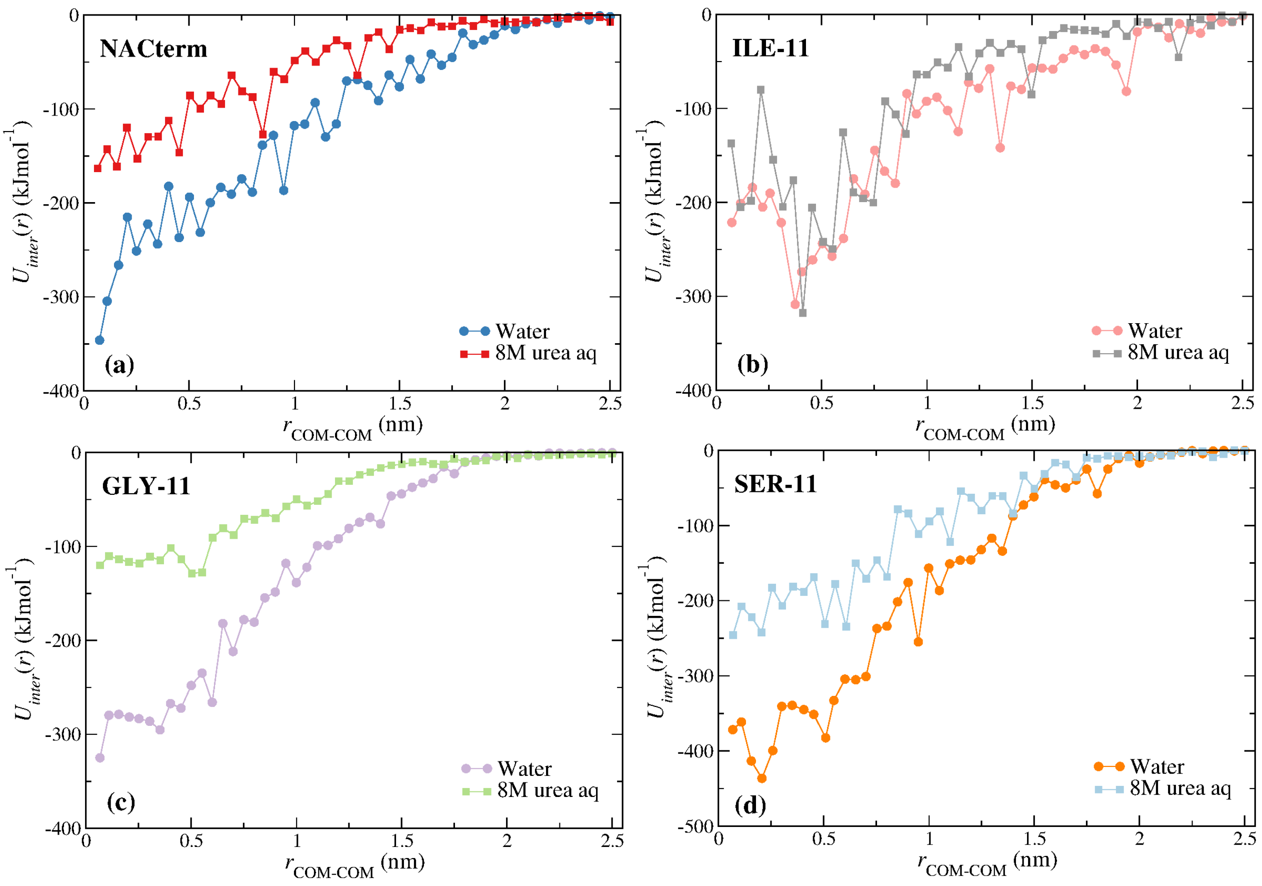

Figure S6 – Interpeptide potential energy profiles in water and 8M aqueous urea solution computed along the umbrella sampling reaction coordinate. Note that, unlike the PMF which represent relative values, the potential energy converges to zero; lines are guide to the eye.

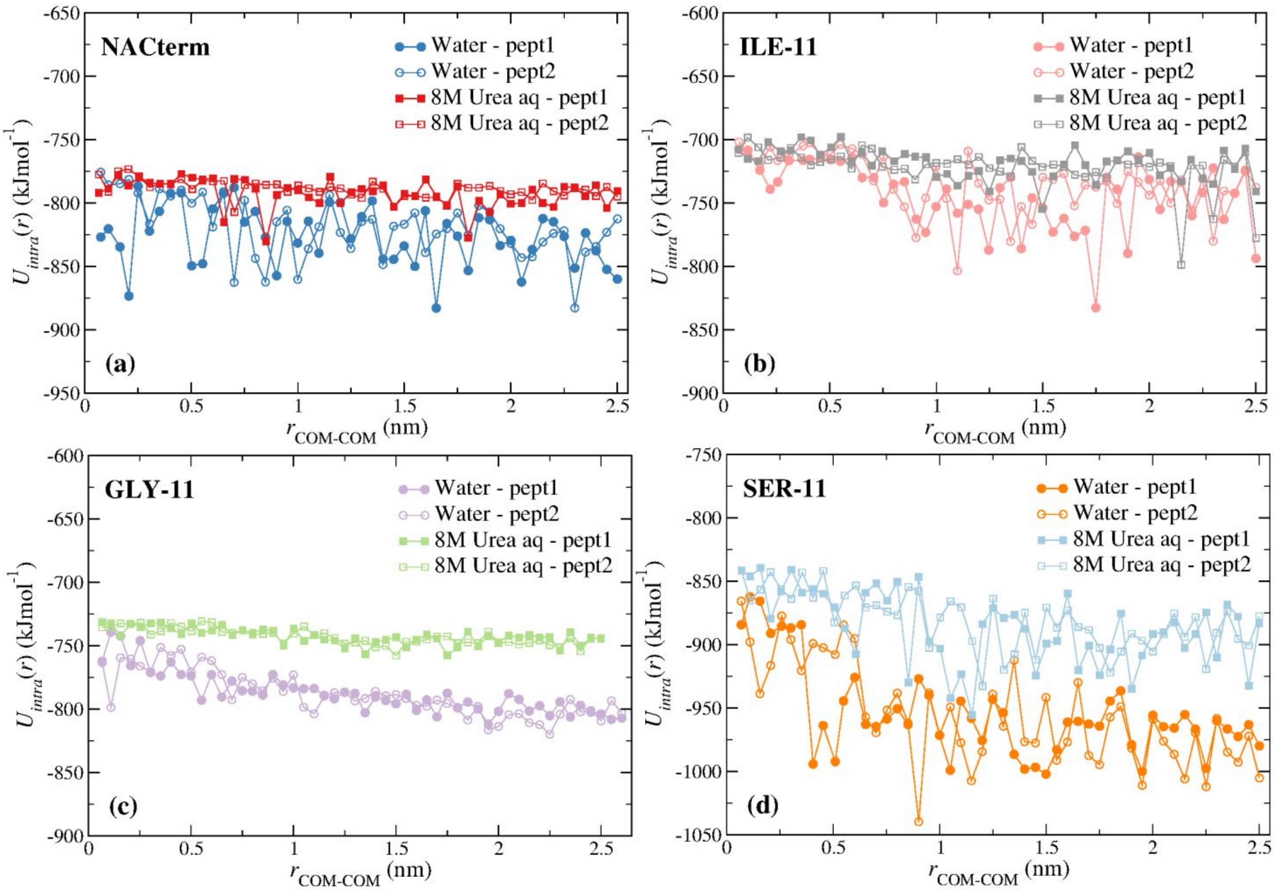

Figure S7 – Intrapeptide potential energy profiles in water and an 8M aqueous urea solution computed along the umbrella sampling reaction coordinate. The *y*-axis spans 300 kJmol^{-1} for every system. Urea's effect is less pronounced in the ILE-11 system, similar to interpeptide interactions.

References

(1) Duarte Ramos Matos, G.; Kyu, D. Y.; Loeffler, H. H.; Chodera, J. D.; Shirts, M. R.; Mobley, D. L. Approaches for Calculating Solvation Free Energies and Enthalpies Demonstrated with an Update of the FreeSolv Database. *Journal of Chemical & Engineering Data* **2017**, *62* (5), 1559–1569. https://doi.org/10.1021/acs.jced.7b00104.

(2) Bennett, C. H. Efficient Estimation of Free Energy Differences from Monte Carlo Data. *Journal of Computational Physics* **1976**, *22* (2), 245–268. https://doi.org/10.1016/0021-9991(76)90078-4.

(3) Ben-Naim, A.; Marcus, Y. Solvation Thermodynamics of Nonionic Solutes. *The Journal of Chemical Physics* **1984**, *81* (4), 2016–2027. https://doi.org/10.1063/1.447824.

(4) Van Gunsteren, W. F.; Berendsen, H. J. C. A Leap-Frog Algorithm for Stochastic Dynamics. *Molecular Simulation* **1988**, *1* (3), 173–185. https://doi.org/10.1080/08927028808080941.

(5) Steinbrecher, T.; Joung, I.; Case, D. A. Soft-Core Potentials in Thermodynamic Integration: Comparing One- and Two-Step Transformations. *J. Comput. Chem.* **2011**, *32* (15), 3253–3263. https://doi.org/10.1002/jcc.21909.

(6) Beutler, T. C.; Mark, A. E.; van Schaik, R. C.; Gerber, P. R.; van Gunsteren, W. F. Avoiding Singularities and Numerical Instabilities in Free Energy Calculations Based on Molecular Simulations. *Chemical Physics Letters* **1994**, *222* (6), 529–539. https://doi.org/10.1016/0009-2614(94)00397-1.

(7) Gapsys, V.; Seeliger, D.; de Groot, B. L. New Soft-Core Potential Function for Molecular Dynamics Based Alchemical Free Energy Calculations. *J. Chem. Theory Comput.* **2012**, *8* (7),

2373–2382. https://doi.org/10.1021/ct300220p.
(8) Pham, T. T.; Shirts, M. R. Identifying Low Variance Pathways for Free Energy Calculations of Molecular Transformations in Solution Phase. *The Journal of Chemical Physics* **2011**, *135* (3), 034114. https://doi.org/10.1063/1.3607597.
(9) Parrinello, M.; Rahman, A. Polymorphic Transitions in Single Crystals: A New Molecular Dynamics Method. *Journal of Applied Physics* **1981**, *52* (12), 7182–7190. https://doi.org/10.1063/1.328693.
